# A randomised trial of ondansetron for the treatment of irritable bowel syndrome with diarrhoea

**DOI:** 10.1136/gutjnl-2013-305989

**Published:** 2013-12-12

**Authors:** Klara Garsed, Julia Chernova, Margaret Hastings, Ching Lam, Luca Marciani, Gulzar Singh, Amanda Henry, Ian Hall, Peter Whorwell, Robin Spiller

**Affiliations:** 1 Nottingham Digestive Diseases Biomedical Research Unit, Queens Medical Centre, Nottingham, UK; 2 Neurogastroenterology Unit, Wythenshawe Hospital, Manchester, UK; 3 Sir Peter Mansfield Magnetic Resonance Imaging Centre, University of Nottingham, Nottingham, UK; 4 Department of Molecular Medicine, School of Surgical and Medical Sciences, University of Nottingham, Nottingham, UK

**Keywords:** Irritable Bowel Syndrome, Serotonin, Clinical Trials

## Abstract

**Background:**

Irritable bowel syndrome with diarrhoea (IBS-D) is particularly debilitating due to urgency and episodic incontinence. Some 5-hydroxytryptamine 3 (5-HT3) receptor antagonists (5-HT3RAs) have proven effective but have serious side effects. Ondansetron, also a 5-HT3RA, has been widely used as an antiemetic with an excellent safety record for over two decades. Our aim was to assess its effectiveness in IBS-D.

**Methods:**

120 patients meeting Rome III criteria for IBS-D entered a randomised, double-blind, placebo-controlled crossover study of 5 weeks of ondansetron 4 mg versus placebo with dose titration allowed, up to two tablets three times daily in the first 3 weeks. Patients completed daily diaries documenting stool consistency using the Bristol Stool Form score. Gut transit was measured in the last week of each treatment. The primary endpoint was average stool consistency in the last 2 weeks of treatment.

**Results:**

Ondansetron significantly improved stool consistency (mean difference in stool form between ondansetron and placebo −0.9, 95% CI −1.1 to −0.6, p<0.001). Compared with placebo, patients on ondansetron experienced fewer days with urgency (p<0.001), lower urgency scores (p<0.001), reduced frequency of defaecation (p=0.002) and less bloating (p=0.002), although pain scores did not change significantly. IBS symptom severity score fell more with ondansetron than placebo (83±9.8 vs 37±9.7, p=0.001). 65% reported adequate relief with ondansetron but not placebo compared with 14% reporting relief with placebo but not ondansetron, relative risk 4.7, 95% CI 2.6 to 8.5, p<0.001.

**Conclusions:**

Ondansetron relieves some of the most intrusive symptoms of IBS-D, namely loose stools, frequency and urgency.

**Significance of this study**

**What is already known about this subject?**

- 5-Hydroxytryptamine 3 receptor antagonists (5-HT3RAs) improve symptoms of irritable bowel syndrome with diarrhoea (IBS-D).
- Alosetron, the best studied 5-HT3RA, was withdrawn from general use because of constipation and rarely ischaemic colitis.
- Ondansetron is a 5-HT3RA widely used as an anti-nauseant for over 20 years and never associated with ischaemic colitis.

**What are the new findings?**

- Ondansetron improves symptoms of frequent loose stools with urgency characteristic of IBS-D.
- Ondansetron slows the accelerated colonic transit associated with IBS-D.
- Those with severe diarrhoea respond less well.

**How might it impact on clinical practice in the foreseeable future**

- Ondansetron is a safe inexpensive 5-HT3RA available worldwide which could improve symptoms in many patients with IBS-D with mild to moderate symptoms.
- The main benefit, which is seen within 7 days, is to reduce urgency.
- The median dose is 4 mg per day in those who respond.

## Introduction, background and objectives

Irritable bowel syndrome with diarrhoea (IBS-D) affects approximately 3% of the general population and accounts for approximately 20% of gastroenterology outpatient visits in the UK. Since many conditions can cause diarrhoea, such patients typically undergo numerous negative tests. IBS regardless of subtype is also associated with considerable impairment of quality of life.^1^ ^2^ IBS-D is particularly a debilitating form of IBS as it reduces the ability to eat out and socialise because of fear of pain, urgent defaecation and even incontinence. Serotonin (5-hydroxytryptamine (5-HT)) is a major mediator in the gut, signalling via afferent nerves to influence gut motility and secretion.^3^ 5-HT3 receptor antagonists (5HT3RAs) block the vagal stimulation induced by 5-HT^4^ and were developed as a highly effective treatment for chemotherapy-induced nausea and vomiting, known to be mediated via vagal stimulation by cisplatinum-induced 5-HT release.^5^ It was soon discovered that 5HT3RAs also cause constipation.^6^ Early studies with ondansetron demonstrated that 16 mg three times a day, the usual dose for chemotherapy-induced emesis, delayed colonic transit in healthy subjects,^7^ and reduced the postprandial increase in colonic tone in carcinoid diarrhoea.^8^ A small trial using the much lower dose of 4 mg three times a day suggested benefit in IBS and functional dyspepsia.^9^ Our aim was to determine whether this inexpensive, safe generic drug would provide similar relief to patients with IBS-D. We also wished to determine the mechanism of action and in particular whether clinical factors or polymorphisms in the SERT genotype could predict those that would respond, as has been suggested for alosetron.^10^

## Methods

### Trial design

**Figure 1:**
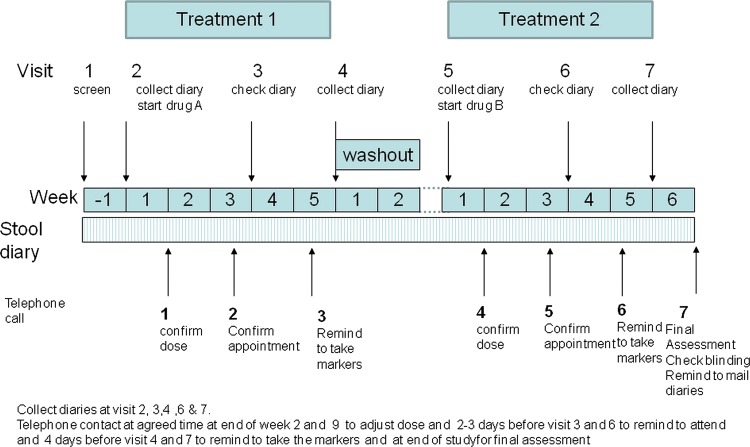
Study design. This shows the two 5-week treatment periods during which subjects were randomised to either ondansetron or placebo. Week 1 was for baseline assessment, Each 5-week treatment period allowed dose adjustment until weeks 4 and 5 when no further dose adjustment or rescue medication was allowed. Symptoms on weeks 4 and 5 provided the clinical endpoints. Symptoms were assessed throughout the study and during the washout period to ensure symptoms had returned to baseline before starting the next treatment. Frequent visits and telephone contact ensured protocol compliance.

This was a two-centre, randomised, double-blind, placebo-controlled crossover study of ondansetron 4 mg/tablet versus placebo. Patients were given one to two tablets three times a day with dose titration for the first 3 weeks of each period and a 2–3-week washout period (figure 1). The trial was registered on clinicaltrials.gov (identifier NCT00745004), approved by Nottingham Research Ethics Committee 2 (REC reference number 08/H0408/134) and by the Medicines and Healthcare Regulatory authority (MHRA, London, UK), and conducted according to Good Clinical Practice guidelines. Funding was provided by the National Institute for Health Research through a Research for Patient Benefit grant and salary support for Dr Garsed from the Nottingham Digestive Diseases Biomedical Research Unit. There were no changes to protocol from that initially registered with clinicaltrials.gov.

### Randomisation

Sequence allocation randomisation was carried out by Nottingham Clinical Trials Support Unit (CTSU) with random permuted blocks of randomly varying size and stratified by centre. The supervising staff obtained a randomisation reference number by a remote, internet-based randomisation system. All participants stayed blinded until the study, data collection and assessments were complete. CTSU Data Manager and the Queen Medical Centre (QMC) Trials pharmacy had access to the treatment allocations. The code was never broken.

### Sample size calculation

From previous studies^11^ the estimated mean (SD) stool consistency in healthy controls was 3.6 (1.1) and recent unpublished data suggest a within-person correlation of 0.5. To detect a difference of 0.4 which was considered clinically signficant with 90% power and 1% type I error we needed 113 subjects. To acount for dropouts we randomised 120.

### Participants

Patients with IBS-D were recruited from IBS clinics at the Queen's Medical Centre Nottingham and Wythenshawe Hospital, Manchester and via the Trent Primary Care Research Network from 1 January 2009 to May 2011 using the Rome III diagnostic criteria.^12^ To exclude other causes of diarrhoea we required a normal colonoscopy and colonic biopsies, normal full blood count, serum calcium and albumen, C-reactive protein and negative serological tests for celiac disease. All patients consuming more than the equivalent of 240 ml of milk/day were tested for lactose intolerance. Most had either a therapeutic trial of colestyramine or a test of bile salt absorption using the 7-day retention of selenium^75^-labelled homocholic acid taurine to exclude bile salt malabsorption. Patients gave written informed consent. Inclusion criteria were age 18–75 years; meeting Rome III criteria; no evidence of inflammatory bowel disease/microscopic colitis and able to give informed consent. Women of child-bearing potential tested negatively on pregnancy test and had to agree to adequate contraception during the study. Patients on selective serotonin reuptake inhibitors or tricyclic antidepressants were included, provided they had been on medication for at least 3 months and the dose remained unaltered throughout the study. Exclusion criteria were pregnancy or breast feeding, unwilling to stop anti-diarrhoeal medication (loperamide or codeine phosphate), prior abdominal surgery other than appendectomy and cholecystectomy, being in another trial or being in the opinion of the investigator unsuitable.

### Healthy controls for transit studies

We also studied 21 healthy controls to provide normal values for the transit studies. They completed the same questionnaires and underwent the same transit measurement protocol. None met Rome III IBS criteria. They comprised 16 women and 5 men, with median age (IQR) of 45 (23–56) years. Bowel frequency was median (IQR) of 1.0 (1.0–1.4) bowel movements per day.

### Intervention

Each participant received 5 weeks of oral placebo treatment and 5 weeks of ondansetron 4 mg tablets using dose titration for the first 3 weeks of each period with a 2–3-week washout period in between each treatment period (figure 1). The hospital pharmacy provided the 5-week drug supply at the beginning of each period. The investigational medicinal product was either a 4 mg ondansetron tablet (Pliva, Zagreb, Croatia) or placebo, both identically over-encapsulated in a gelatine capsule by Bilcare (Crickhowell, Powys, UK). The placebo formulation matched that of the ondansetron in appearance and composition, except for the active drug.

The patients were instructed to start with one capsule once a day, increasing daily to a maximum of two capsules three times a day, depending on the response. If stool consistency increased to stool form 1 or 2, or if bowel frequency dropped below one per day the dose was reduced to a minimum of one capsule taken every 2 days. Patients were required to stay on a stable dose for the last 2 weeks of each period. Loperamide, 2 mg twice daily, was allowed as rescue medication in the event of uncontrolled diarrhoea, but needed to be discontinued for the final 2 weeks of each period. Patients attended for a total of seven visits as follows (figure 1): screening visit 1 was followed by a 1-week period when stool and symptom diaries were completed (week 1). At visit 2, after checking patients met Rome III criteria and had completed the stool diaries, they were enrolled and instructed to start treatment at one capsule daily increasing or decreasing the dose by one capsule per day every 2 days to a maximum of two capsules three times daily. They were told to increase the dose if the stool form was 6 or 7 and decrease if it was 2 or 1. After 1 week patients were telephoned to confirm the dose was optimum. They were also called a few days before visit 3 to confirm the appointment and before visit 4 to remind them to take the transit markers for 3 days before the visit. Visit 3 was after 3 weeks of treatment and visit 4 was the final visit of the first treatment period when stool diaries were collected and colonic transit assessed by a plain X-ray. There was then a 2-week washout which was extended if necessary to ensure bowel habit had returned to baseline. Return to baseline was confirmed by asking patients whether their bowel dysfunction was back to pre-study levels and this was objectively corroborated by examination of the visit 5 diaries. In those participants in whom pre-study levels had not been reached by 2 weeks, initiation of the second treatment phase was delayed until pre-study levels were confirmed.

The second treatment period was identical to the first. Compliance was monitored by asking the patient at each study visit and by a final pill count of all returned medicines.

### Data collection

Personal baseline data were collected at visit 1: age, gender, depression and anxiety scores from the Hospital and Depression Scale, score from the Patient Health Questionnaire 15,^13^ perceived stress score from the Perceived Stress Scale Questionnaire.^14^ IBS-related quality of life from the IBS Quality of Life Questionnaire^15^ and IBS severity score from the IBSS Severity Score Questionnaire^16^ were collected at visit 1 and at the end of each treatment period (visits 4 and 7). We used our previously described daily stool diary^11^ throughout the study to provide information on stool form (Bristol Stool Form score,^17^ from 1 (very hard) to 7 (water)) and pain perception, urgency of defecation and bloating, the last three scored as none, mild, moderate or severe (0–3). Frequency of defaecation and number of days when pain, urgency or bloating was present were recorded.

The baseline values are averages from the screening week.

### Endpoints

The primary endpoint (stool form) and secondary endpoints (pain perception, urgency of defaecation, bloating, frequency of defaecation per day, number of days per week with pain, urgency or bloating, and IBS SS) are averages over the last 2 weeks of each treatment period. Information provided for less than 10 days (out of 14) was recorded as missing.

At the end of each period patients were asked: ‘over the last 2 weeks did you obtain adequate relief of your IBS symptoms?’, and at the end of the study: ‘which treatment if any did you prefer?’, and ‘which treatment, if any would you prefer to continue with now the trial has finished?’ Percentage reporting adequate relief of IBS symtpoms (yes/no), proportion of patients preferring particular treatment (yes/no) and proportion wanting to continue with particular treatment (yes/no) were recorded as secondary endpoints.

Bloods were collected for genetic analysis for the serotonin transporter promoter polymorphism (see online supplementary appendix for methods and results).

### Responder definition

We used the US Food and Drug Administration (FDA) definition of ‘a Stool Consistency Responder’ as a ‘patient who experiences a 50 percent or greater reduction in the number of days per week with at least one stool that has a consistency of Type 6 or 7 compared with baseline’ and a ‘pain responder’ as a patient who experienced a fall of 30% in pain compared to baseline.^18^

The FDA recommends that for IBS a dual endpoint should be used to define a ‘responder’ who should be both a Stool Consistency responder and a ‘pain responder’.

### Whole gut transit measurement

We used the Metcalf's radio-opaque marker technique.^19^ Subjects took 20 silicon markers impregnated with 13.5% barium (Altimex, Nottingham, UK) at 09:00 each morning for three consecutive days. The number identified on plain abdominal X-ray taken on the morning of day 4 was multiplied by 1.2 to give whole gut transit time (WGTT) in hours. Regional transit was assessed from the number of pellets assigned to the ascending colon, transverse, descending and rectosigmoid as described by Metcalf and colleagues.^19^

## Statistical methods

### Efficacy parameters

Baseline values were only available for the screening phase so the efficacy parameters (except response) were calculated for each patient as the differences between the endpoints measured in ondansetron and placebo periods. Frequencies were compared as ratios and treatment effect expressed as percenatges.

### Analysis

Analysis was carried out with Stata 12. First, intention-to-treat analysis (ITT) was carried out with available data. Second, the data were re-analysed as per protocol (PPA). Baseline variables were analysed by dropout status with t test, Kruskal–Wallis test or χ^2^ test for symmetrical, skewed or categorical variables correspondingly.

The continuous efficacy parameters were approximately symmetrical and were analysed with linear regression. Preference and response data were analysed with multinomial logistic regression. The results were not adjusted for multiple testing.

## Results

### Participant flow

**Figure 2:**
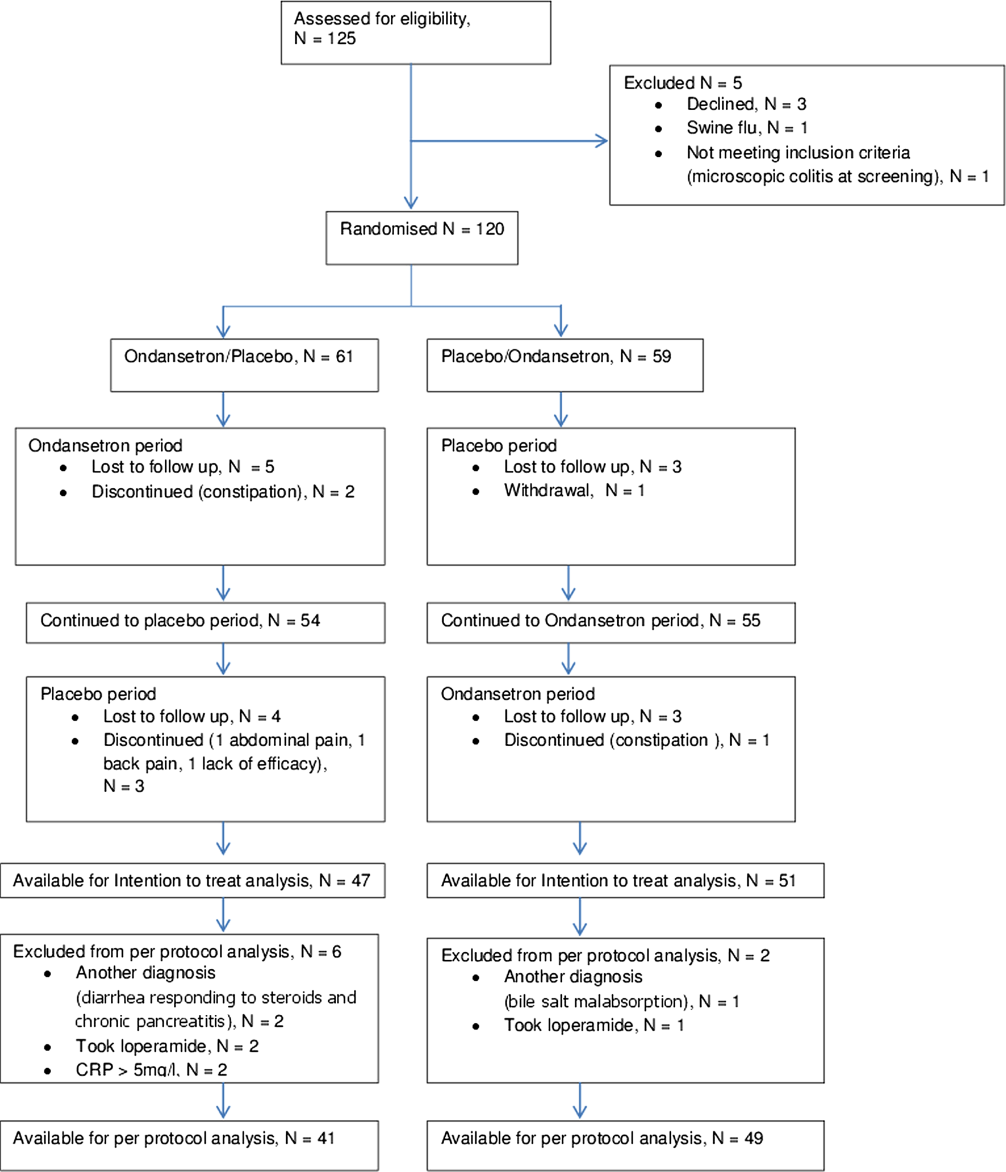
Consort diagram showing patient flow with dropouts and protocol violations. CRP, C-reactive protein.

Of the 125 patients recruited, 120 were randomised as 5 did not complete the screening phase. The CONSORT diagram (figure 2) summarises the flow. There were 47 (77%) patients with ondansetron/placebo sequence and 51 (88%) patients with placebo/ondansetron sequence, giving 98 (82%) patients available for ITT analysis. Nearly twice as many patients dropped out from the ondansetron/placebo arm compared with placebo/ondansetron (p=0.110), mostly during the placebo period (risk ratio for dropping out when starting with ondansetron is 1.9, 95% CI 0.8 to 4.5). Those who dropped out had more bloating and more frequent need to go to toilet (table 1). Ninety (75%) patients were available for PPA.

**Table 1:** Baseline characteristics of patients compared with those who dropped out

	Available for analysis (N=98)	Dropped out (N=22)	p Value (difference between analysed and dropped out)
Age	41 (12)	40 (12)	0.689
Gender (women), N (%)	74 (75.0)	14 (64.0)	0.303
Stool form	5.4 (0.7)	5.5 (0.7)	0.758
Days with morning rush*, median (IQR)	1 (0–3)	2 (0–6)	0.078
Days with pain, median (IQR)	6 (3–7)	6.5 (5–7)	0.256
Days with urgency, median (IQR)	6.5 (5–7)	7 (6–7)	0.642
Days with bloating, median (IQR)	6 (3–7)	7 (5–7)	0.252
Pain score	1.3 (0.8)	1.5 (0.8)	0.574
Urgency score	1.6 (0.7)	1.6 (0.8)	0.826
Bloating score	1.3 (0.9)	1.7 (0.9)	0.085
Frequency of defaecation per day, median (IQR)	2.6 (1.9–3.7)	3.6 (2.1–6.6)	0.033
Anxiety	9.5 (4.3)	10.4 (5.5)	0.553
Depression, median (IQR)	5 (3–9)	5.5 (1–10)	0.670
Patient Health Questionnaire 15	12 (4.0)	13.3 (5.0)	0.283
IBS severity score	302 (85)	330 (83)	0.324
Patient stress score	17.9 (7.4)	20.3 (8.8)	0.322
IBS QOL	455 (150)	403 (189)	0.102

Means (SDs) are shown if not stated otherwise.

*Defined as more than three bowel movements before midday.

IBS, irritable bowel syndrome; QOL, quality of life.

### Primary efficacy parameter

The difference in stool form between ondansetron and placebo was −0.9, 95% CI −1.1 to −0.6, p<0.001, which showed a significant improvement when taking ondansetron compared with placebo. Worse diarrhoea at baseline was associated with decreased effect of ondansetron (every 1 point baseline average stool form increase reduced effectiveness by 0.4 points, 95% CI 0.00 to 0.8, p=0.032). For example, people with less severe diarrhoea (lower quartile: average stool form 4.9) benefited more from ondansetron (stool form difference −1.0, 95% CI −1.3 to −0.7, p<0.001) compared with those with more severe diarrhoea (upper quartile: average stool form 5.9), with stool form difference −0.7, 95% CI −1.0 to −0.4, p<0.001.

PPA showed average −0.9, 95% CI −1.2 to −0.6 stool form difference between ondansetron and placebo. One point increase in baseline stool form was associated with decreased effect of ondansetron by 0.5 points, 95% CI 0.1 to 0.8, p=0.017.

**Figure 3:**
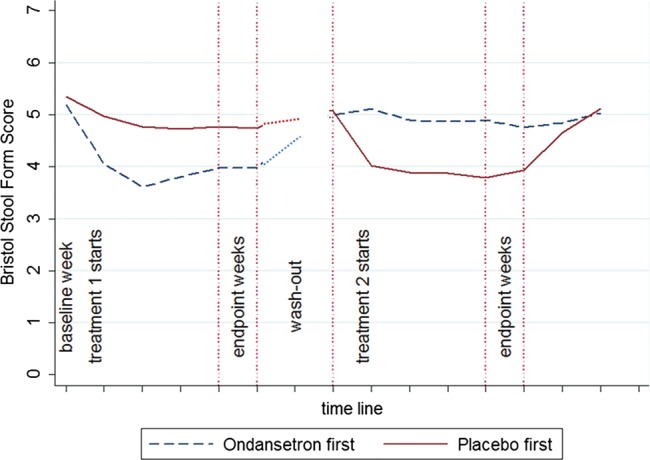
Time course for stool consistency during the two treatment periods. Time shown in weeks. Washout period was variable so the value during the last 7 days is shown as the first data point in the treatment period 2. The graph shows very little placebo effect with rapid onset of treatment effect on commencing ondansetron and loss of effect on discontinuing.

One concern about crossover design studies is the possibility of a carryover effect such that those who received active treatment first would have less symptoms at the beginning of the second treatment period. However the washout period of 2 weeks was sufficient for most patients to report their symptoms were back to baseline and only 17 (17%) needed longer, the maximum period being 36 days. They were asked whether their bowel dysfunction was back to its usual pre-study level and this was confirmed objectively from their symptom diaries on visit 5. Average stool consistency in the week prior to starting the second arm of the trial was slightly improved at 5.2 compared with 5.4 for baseline (p=0.031) but there was no difference according to whether active was first or second (Figure 3). Furthermore, with respect to either average bloating, urgency or abdominal pain scores, there was no difference in the last 7 days of the washout period between those who had active or placebo first, the differences being 0.17 (0.2), 0.01 (0.2) and 0.10 (0.2), p=0.4, 0.7 and 0.6, respectively. Thus symptoms at the start of the second period were not affected by the treatment allocation in the first phase (Figure 3). As can be seen, there was very little placebo response and onset of treatment effect and loss of effect on discontinuing ondansetron was rapid, occurring within the first week in both cases. The median (IQR) dose was 4 (2–6.5) mg for responders and 8 (4–20) mg for non-responders for stool consistency.

### Secondary efficacy parameters

Table 2 shows that in the ITT analysis the number of days with pain and average pain score did not change on ondansetron but patients experienced significantly fewer days with urgency and bloating. Average urgency scores and average frequency of defaecation were significantly lower compared with placebo, though the fall in average bloating scores did not achieve statistical significance. Baseline characteristics did not correlate with the above efficacy parameters. The results were similar for PPA. IBS SSS fell compared with baseline when taking ondansetron by 83±9.8 points, significantly more than the 37±9.7-point fall on placebo, p=0.001. A fall of 50 points in the IBS SSS is regarded as clinically significant.

**Table 2:** Ondansetron effect on secondary outcomes

	ITT analysis (N=98)		PPA (N=90)	
	Treatment effect (95% CI)	p Value	Treatment effect (95% CI)	p Value
Days per week with pain	−0.3 (−0.7 to 0.1)	0.203	−0.3 (−0.7 to 0.2)	0.227
Days per week with urgency	−1.1 (−1.5 to −0.6)	<0.001	−1.1 (−1.6 to −0.7)	<0.001
Days per week with bloating	−0.7 (−1.1 to −0.3)	0.002	−0.7 (−1.1 to −0.3)	0.002
Pain score (0–3) None (0), mild (1), moderate (2) or severe (3)	−0.10 (−0.22 to 0.03)	0.119	−0.10 (−0.23 to 0.02)	0.103
Urgency score(0–3) None (0), mild (1), moderate (2) or severe (3)	−0.32 (−0.45 to −0.18)	<0.001	−0.33 (−0.47 to −0.19)	<0.001
Bloating score(0–3) None (0), mild (1), moderate (2) or severe (3)	−0.13 (−0.27 to 0.01)	0.070	−0.12 (−0.26 to 0.02)	0.103
Stool frequency reduction, %	11 (4 to 18)	0.001	11 (4 to 18)	0.002
Whole gut transit time increase*, h	10 (6 to 14)	<0.001	10 (6 to 14)	<0.001
Right colon transit time increase, h	2 (0 to 4)	0.064	2 (0 to 4)	0.082
Left colon transit time increase, h	6 (3 to 8)	<0.001	5 (3 to 8)	<0.001

Differences between ondansetron and placebo are presented.

*Numbers of patients available for analysis are 87/98 (89%) for ITT and 81/90 (90%) for PPA analysis. Lower numbers reflect patients who failed to take their markers or attend for the final X-ray.

ITT, intention-to-treat; PPA, per protocol analysis.

Using the FDA criteria, 80% of patients responded with a reduction in number of days with loose stools while taking ondansetron compared with 41% on placebo. The FDA criteria for pain were met by 43% on ondansetron and 40% on placebo and the combined FDA criteria were met by 41% on ondansetron and 17% on placebo. Preference data were available only for a subsample of patients (N=94 for ITT and N=86 for PPA). Table 3 shows the preference distribution. A significantly higher proportion of patients prefer, would continue with and had adequate relief with ‘ondansetron but not placebo’ compared with ‘placebo but not ondansetron’ (all p<0.001).

**Table 3:** Patient preferences and true stool responder data

	Ondansetron No Placebo Yes	Ondansetron Yes Placebo No	Ondansetron No Placebo No	Ondansetron Yes Placebo Yes	Risk ratio to prefer ‘ondansetron but not placebo’ compared with ‘placebo but not ondansetron’
ITT (N=94)					
Preference, N (%)	15 (16)	70 (74)	9 (10)	0 (0)	4.7 (2.7 to 8.2)
p value					<0.001
Continue, N (%)	15 (16)	67 (71)	11 (12)	1 (1)	4.5 (2.6 to 7.8)
p value					<0.001
Adequate Relief, N (%)	13 (14)	61 (65)	17 (18)	3 (3)	4.7 (2.6 to 8.5)
p value					<0.001
PPA (N=86)					
Preference, N (%)	13 (15)	66 (77)	7 (8)	0 (0)	5.1 (2.8 to 9.2)
p value					<0.001
Continue, N (%)	14 (16)	64 (74)	7 (8)	1 (1)	4.6 (2.6 to 8.2)
p value					<0.001
Adequate Relief, N(%)	11 (13)	58 (67)	14 (16)	3 (3)	5.3 (2.8 to 10.0)
p value					<0.001
*Response data*					
ITT (N=98)					
True stool responder					
Response, N (%)	6 (6)	44 (45)	14 (14)	34 (35)	7.3 (3.1 to 17.2)
p value					<0.001
PPA (N=90)					
Response, N (%)	5 (6)	43 (48)	11 (12)	31 (34)	8.6 (3.4 to 21.7)
p value					<0.001

*Ratios are shown for probability that people would prefer a particular choice compared with the choice ‘Placebo yes but ondansetron no’. For example, it is much more probable (4.2 times, 95% CI 2.5 to 7.1) that patients prefer ‘Ondansetron but not placebo’ compared with ‘Placebo but not ondansetron’. The same applies to ‘continue’ and ‘adequate relief’ options. Data show that it is more probable (7.3 times, 95% CI 3.1 to 17.2) to respond to ondansetron but not placebo compared with placebo but not ondansetron.

### Gut transit time

Gut transit time (table 4) was available for 87 patients in the ITT analysis and 81 patients in the PPA. Both showed significantly longer values for ondansetron compared with placebo, with differences of 10 h, 95% CI 6 to 14 h, p<0.001. Patients with IBS-D on placebo showed significantly faster transit, with values of 16 (7 to 29) h compared with 46 (12 to 58) h for healthy controls. Regional transit times are given in table 4, showing that the most marked difference was in the faster transit through the left colon and rectosigmoid, something which ondansetron tended to correct, shifting transit towards the normal range so that transit times were no longer significantly different from controls. We found no difference in this effect between the three SERT promoter polymorphisms, though there was a tendency for the *sl* genotype to be associated with a greater clinical effect and the WGTT increase was 17.1 (10.6 to 23.7) for the *sl* genotype compared with 4.9 (−3.0 to 12.8) for *ll*, ANOVA, p=0.07 (see online supplementary appendix 1).

**Table 4:** Whole gut and regional transit times (median (IQR))

	Whole gut transit time	Right colon	Left colon	Rectosigmoid
Healthy controls N=19	46 (12–58)	13 (5–18)	12 (3–24)	7 (4–15)
Patients with IBS-D on placebo	16 (7–29)*	6 (2–12)	2.5 (0–7)***	4 (1–9)***
Patients with IBS-D on ondansetron	24 (15–47)	7 (3–16)	6 (0–17.25)	7 (2–13)
P for difference on ondansetron		NS	<0.05	<0.05

*p<0.05, ***p<0.001 versus healthy volunteers.

IBS-D, irritable bowel syndrome with diarrhoea.

### Adverse events

The only frequently occurring side effect was constipation, which occurred in 9%^10^ on ondansetron and 2%^2^ on placebo. All responded to dose reduction and only two decided to leave the trial at that point. Other less frequent side effects included headache (2 ondansetron, 2 placebo), rectal bleeding (2 ondansetron, 2 placebo, none of which were found to be due to ischaemic colitis), backache (1 ondansetron, 1 placebo) and abdominal pain (2 ondansetron, 1 placebo).

## Discussion

Patients with IBS-D suffer markedly from loose and frequent stools, and particularly from the associated urgency and fear of incontinence which generates panic and anxiety. This, therefore represents an important unmet need. The abnormalities of serotonin metabolism which have been demonstrated in IBS-D make 5-HT3 antagonists a logical treatment. Post-infective IBS, a subtype of IBS-D with very similar clinical features,^20^ has been shown to be associated with increased 5HT-containing enteroendocrine cells,^21–23^ and also increased postprandial 5-HT release.^24^ Further studies have also shown reduced mRNA for SERT in IBS-D duodenal^25^ and colonic biopsies^23^ ^25^which, in keeping with animal studies of post inflammatory bowel dysfunction,^26^ was correlated with mucosal immune response.

Our study showed patients with IBS-D have a clear preference for ondansetron compared with placebo, even though it did not alter the number of days with pain, suggesting that for these patients it was urgency and loose stools which were the most troublesome symptoms. It is of interest that animal studies have shown that alosetron, a 5-HT3RA shown to be effective in IBS-D, inhibits spinal pathways mediating the response to painful colonic distension.^27^ We have also shown in a rat model of postinfective visceral hypersensitivity that ondansetron reduces afferent firing induced by colonic distension,^26^ suggesting that ondansetron might have reduced pain if we had used higher doses. However, this would undoubtedly have produced more constipation which our patients were keen to avoid. Although only 41% met FDA criteria for responder to both pain and consistency, 67% reported adequate relief of symptoms, suggesting that when urgency is the main problem ondansetron will be effective but less so when pain is the main complaint. It should be noted that dyspepsia is a common comorbidity with IBS and earlier studies suggest benefit to heartburn and postprandial pain.^9^ Therefore some of the global benefit may have been from improvement in dyspepsia, though we did not assess this in the current study. Future studies should probably include a dyspepsia assessment.

Our variable dosing regime mirrored clinical practice and we feel gives a better idea of how the drug will perform in clinical practice. It undoubtedly improved response rate and had we chosen a fixed dose many patients would have developed constipation and probably dropped out or had worsening symptoms. As expected, the median number of tablets per day was much lower for ondansetron than placebo, median (IQR) 1 (0.5–1.4) and 2 (1–5), respectively. However, dose titration does complicate analysis since only the stool diaries in the last 2 weeks are truly informative of the response, meaning that we cannot impute data to allow for incomplete diaries, which would be normal practice. These incomplete data led to only 98 subjects being analysed.

Our evaluation time of just 2 weeks was short but in patients with diarrhoea and rapid transit, drug effects tend to be rapid and quickly reversible, for example with loperamide. We found that most subjects returned to their previous bowel habit within the 2-week washout and only 17 patients required longer.

Our patients were moderately affected by bowel disturbance and psychological comorbidity similar to other recent trials of IBS-D treatment,^28^ so we expect our findings to be generalisable to the general IBS population with symptoms severe enough to warrant referral to secondary care.

Regrettably alosetron, a 5-HT3RA for which there is substantial evidence of benefit,^29^ was withdrawn from widespread use because of an unacceptable incidence of severe constipation (around 25%) and a much lower incidence (0.7 per 1000 patient-years^30^) of unexplained ischaemic colitis. Our trial shows that ondansetron can achieve useful results with a low incidence of side effects. While our small study cannot prove safety, the fact that the drug has been used widely for over 25 years without a single report of ischaemic colitis suggests that this side effect will be rare. Constipation occurred in just 9% and rapidly resolved on dose reduction, giving a discontinuation rate due to constipation of just 2%. Our constipation rate is lower than the 18–29.6% and 10–19.3% respectively reported in the meta-analysis of alosetron 2 mg and cilansetron 6 mg daily,^29^ while ramosetron 5 μg was associated with a comparable rate of 7.4%.^31^ Our low rate undoubtedly reflects the use of dose titration, to avoid constipation, with many patients only requiring 4 mg daily or on alternate days. This mirrors what would happen in clinical practice. The high incidence of constipation with alosetron is dose related and 0.5 mg daily is associated with a much reduced incidence of constipation (just 9%) with relief of symptoms in 50.8% of patients compared with 30.7% in placebo.^32^ Unfortunately ischaemic colitis remains a concern even at 0.5mg daily despite careful monitoring, with an incidence of 2 per 1000 with alosetron^33^ and it seems unlikely that alosetron will ever be marketed worldwide. Ondansetron's potency in blocking the 5-HT3 receptor is 3–10 times lower than alosetron,^34^ which may explain the low incidence of side effects in our study. Ramosetron is another 5-HT3RA, proven effective in IBS-D,^35^ but unfortunately only marketed in Japan. It has an affinity for the 5-HT3 receptor three times that of alosetron^36^ but is given at a very low dose of 5 μg, equivalent to 0.015 mg alosetron, again suggesting that lower doses of 5-HT3RAs might well be the best strategy in treating IBS-D.

Previous authors have reported that individuals with the heterozygote *sl* genotype responded less well to alosetron as assessed by the change in colonic transit,^10^ a finding which does not seem true for ondansetron (see online supplementary appendix).

The strongest effects were on transit, stool consistency and urgency, which are important since urgency is one of the strongest predictors of reduced quality of life^37^ and as others have reported, response to 5-HT3RAs also correlates with improvement in quality of life.^38^

Unlike the larger alosetron trials we did not find a significant improvement in abdominal pain versus placebo, with only 41% of individuals meeting FDA dual criteria for being ‘responders’ for pain and stool consistency, a value not significantly different from 17% on placebo. However, 67% of our patients reported ‘adequate relief’ from their symptoms with ondansetron but not placebo, compared with 14% with placebo but not ondansetron. Furthermore, the IBS SSS score, an overall IBS severity score, fell significantly compared with placebo. The largest numerical effect we found was the reduction in days with urgency, which is known to be one of the most bothersome symptoms in IBS-D^1^ and a very important driver of impairment of quality of life.^39^ It is also rated by patients as the most important attribute of a successful treatment for IBS-D.^40^

We found that those most severely affected were more likely to drop out, less likely to respond and showed a smaller reduction in stool consistency, indicating that the efficacy for treating severe diarrhoea is limited and the best response will be in those with mild to moderate symptoms who represent the majority of patients seen in primary care. However, given its safety, low side effect profile and rapid onset of effect within 1 week in most cases, a trial of treatment would seem reasonable in most cases of IBS-D. Whether it would help patients with functional diarrhoea remains to be determined.

Ondansetron is a generic drug, available worldwide at a low price, with a very long experience of safe usage, which our study suggests would benefit patients with IBS-D troubled mainly by urgency and loose stools.

## Supplementary Material

Web appendix

## References

[1] Tillisch K Labus JS Naliboff BD Characterization of the alternating bowel habit subtype in patients with irritable bowel syndrome. Am J Gastroenterol 2005;100:896–904 15784038 10.1111/j.1572-0241.2005.41211.x

[2] Spiegel BM Gralnek IM Bolus R Clinical determinants of health-related quality of life in patients with irritable bowel syndrome. Arch Intern Med 2004;164:1773–80 15364671 10.1001/archinte.164.16.1773

[3] Gershon MD Review article: serotonin receptors and transporters—roles in normal and abnormal gastrointestinal motility. Aliment Pharmacol Ther 2004;20 (Suppl 7):3–14 15521849 10.1111/j.1365-2036.2004.02180.x

[4] Zhu JX Zhu XY Owyang C Intestinal serotonin acts as a paracrine substance to mediate vagal signal transmission evoked by luminal factors in the rat. J Physiol 2001;530 (Pt 3):431–42 11158274 10.1111/j.1469-7793.2001.0431k.x PMC2278417

[5] Wolf H Preclinical and clinical pharmacology of the 5-HT3 receptor antagonists. Scand J Rheumatol Suppl 2000;113:37–45 11028830 10.1080/030097400446625

[6] Cremonini F Delgado-Aros S Camilleri M Efficacy of alosetron in irritable bowel syndrome: a meta-analysis of randomized controlled trials. Neurogastroenterol Motil 2003;15:79–86 12588472 10.1046/j.1365-2982.2003.00389.x

[7] Talley NJ Phillips SF Haddad A GR 38032F (ondansetron), a selective 5HT3 receptor antagonist, slows colonic transit in healthy man. Dig Dis Sci 1990;35:477–80 2138532 10.1007/BF01536922

[8] von der Ohe MR Camilleri M Kvols LK A 5HT3 antagonist corrects the postprandial colonic hypertonic response in carcinoid diarrhea. Gastroenterology 1994;106:1184–9 8174881 10.1016/0016-5085(94)90008-6

[9] Maxton DG Morris J Whorwell PJ Selective 5-hydroxytryptamine antagonism: a role in irritable bowel syndrome and functional dyspepsia? Aliment Pharmacol Ther 1996;10:595–9 8853764 10.1046/j.1365-2036.1996.30172000.x

[10] Camilleri M Atanasova E Carlson PJ Serotonin-transporter polymorphism pharmacogenetics in diarrhea-predominant irritable bowel syndrome. Gastroenterology 2002;123:425–32 12145795 10.1053/gast.2002.34780

[11] Marciani L Cox EF Hoad CL Postprandial changes in small bowel water content in healthy subjects and patients with irritable bowel syndrome. Gastroenterology 2010;138:469–77 19909743 10.1053/j.gastro.2009.10.055

[12] Longstreth GF Thompson WG Chey WD Functional bowel disorders. Gastroenterology 2006;130:1480–91 16678561 10.1053/j.gastro.2005.11.061

[13] Kroenke K Spitzer RL Williams JB The PHQ-15: validity of a new measure for evaluating the severity of somatic symptoms. Psychosom Med 2002;64:258–66 11914441 10.1097/00006842-200203000-00008

[14] Levenstein S Prantera C Varvo V Development of the Perceived Stress Questionnaire: a new tool for psychosomatic research. J Psychosom Res 1993;37:19–32 8421257 10.1016/0022-3999(93)90120-5

[15] Hahn BA Kirchdoerfer LJ Fullerton S Evaluation of a new quality of life questionnaire for patients with irritable bowel syndrome. Aliment Pharmacol Ther 1997;11:547–52 9218081 10.1046/j.1365-2036.1997.00168.x

[16] Francis CY Morris J Whorwell PJ The irritable bowel severity scoring system: a simple method of monitoring irritable bowel syndrome and its progress. Aliment Pharmacol Ther 1997;11:395–402 9146781 10.1046/j.1365-2036.1997.142318000.x

[17] Heaton KW O'Donnell LJ An office guide to whole-gut transit time. Patients’ recollection of their stool form. J Clin Gastroenterol 1994;19:28–30 7930429 10.1097/00004836-199407000-00008

[18] US Department of Health and Human Services, Food and Drug Administration, Center for Drug Evaluation and Research. Guidance for industry irritable bowel syndrome—clinical evaluation of drugs for treatment. 2012 Silver Spring, MD: Office of Communications, Division of Drug Information Center for Drug Evaluation and Research Food and Drug Administration

[19] Metcalf AM Phillips SF Zinsmeister AR Simplified assessment of segmental colonic transit. Gastroenterology 1987;92:40–7 3023168 10.1016/0016-5085(87)90837-7

[20] Spiller R Garsed K Postinfectious irritable bowel syndrome. Gastroenterology 2009;136:1979–88 19457422 10.1053/j.gastro.2009.02.074

[21] Spiller RC Jenkins D Thornley JP Increased rectal mucosal enteroendocrine cells, T lymphocytes, and increased gut permeability following acute Campylobacter enteritis and in post-dysenteric irritable bowel syndrome. Gut 2000;47:804–11 11076879 10.1136/gut.47.6.804 PMC1728147

[22] Dunlop SP Jenkins D Neal KR Relative importance of enterochromaffin cell hyperplasia, anxiety, and depression in postinfectious IBS. Gastroenterology 2003;125:1651–9 14724817 10.1053/j.gastro.2003.09.028

[23] Faure C Patey N Gauthier C Serotonin signaling is altered in irritable bowel syndrome with diarrhea but not in functional dyspepsia in pediatric age patients. Gastroenterology 2010;139:249–58 20303355 10.1053/j.gastro.2010.03.032 PMC2902614

[24] Dunlop SP Coleman NS Blackshaw E Abnormalities of 5-hydroxytryptamine metabolism in irritable bowel syndrome. Clin Gastroenterol Hepatol 2005;3:349–57 15822040 10.1016/s1542-3565(04)00726-8

[25] Foley S Garsed K Singh G Impaired uptake of serotonin by platelets from patients with irritable bowel syndrome correlates with duodenal immune activation. Gastroenterology 2011;140:1434–43 21315720 10.1053/j.gastro.2011.01.052

[26] Keating C Beyak M Foley S Afferent hypersensitivity in a mouse model of post-inflammatory gut dysfunction: role of altered serotonin metabolism. J Physiol 2008;586 (Pt 18):4517–30 18653657 10.1113/jphysiol.2008.156984 PMC2614020

[27] Kozlowski CM Green A Grundy D The 5-HT(3) receptor antagonist alosetron inhibits the colorectal distention induced depressor response and spinal c-fos expression in the anaesthetised rat. Gut 2000;46:474–80 10716675 10.1136/gut.46.4.474 PMC1727898

[28] Brown PM Drossman DA Wood AJ The tryptophan hydroxylase inhibitor LX1031 shows clinical benefit in patients with nonconstipating irritable bowel syndrome. Gastroenterology 2011;141:507–16 21684281 10.1053/j.gastro.2011.05.005 PMC4905727

[29] Andresen V Montori VM Keller J Effects of 5-hydroxytryptamine (serotonin) type 3 antagonists on symptom relief and constipation in nonconstipated irritable bowel syndrome: a systematic review and meta-analysis of randomized controlled trials. Clin Gastroenterol Hepatol 2008;6:545–55 18242143 10.1016/j.cgh.2007.12.015 PMC2587294

[30] Chang L Chey WD Harris L Incidence of ischemic colitis and serious complications of constipation among patients using alosetron: systematic review of clinical trials and post-marketing surveillance data. Am J Gastroenterol 2006;101:1069–79 16606352 10.1111/j.1572-0241.2006.00459.x

[31] Matsueda K Harasawa S Hongo M A phase II trial of the novel serotonin type 3 receptor antagonist ramosetron in Japanese male and female patients with diarrhea-predominant irritable bowel syndrome. Digestion 2008;77:225–35 18667823 10.1159/000150632

[32] Krause R Ameen V Gordon SH A randomized, double-blind, placebo-controlled study to assess efficacy and safety of 0.5 mg and 1 mg alosetron in women with severe diarrhea-predominant IBS. Am J Gastroenterol 2007;102:1709–19 17509028 10.1111/j.1572-0241.2007.01282.x

[33] Chang L Tong K Ameen V Ischemic colitis and complications of constipation associated with the use of alosetron under a risk management plan: clinical characteristics, outcomes, and incidences. Am J Gastroenterol 2010;105:866–75 20197759 10.1038/ajg.2010.25

[34] Clayton NM Sargent R Butler A The pharmacological properties of the novel selective 5-HT3 receptor antagonist, alosetron, and its effects on normal and perturbed small intestinal transit in the fasted rat. Neurogastroenterol Motil 1999;11:207–17 10354345 10.1046/j.1365-2982.1999.00148.x

[35] Matsueda K Harasawa S Hongo M A randomized, double-blind, placebo-controlled clinical trial of the effectiveness of the novel serotonin type 3 receptor antagonist ramosetron in both male and female Japanese patients with diarrhea-predominant irritable bowel syndrome. Scand J Gastroenterol 2008;43:1202–11 18618371 10.1080/00365520802240255

[36] Hirata T Keto Y Funatsu T Evaluation of the pharmacological profile of ramosetron, a novel therapeutic agent for irritable bowel syndrome. J Pharmacol Sci 2007;104:263–73 17652911 10.1254/jphs.fp0070620

[37] Wiklund IK Fullerton S Hawkey CJ An irritable bowel syndrome-specific symptom questionnaire: development and validation. Scand J Gastroenterol 2003;38:947–54 14531531 10.1080/00365520310004209

[38] Cremonini F Nicandro JP Atkinson V Randomised clinical trial: alosetron improves quality of life and reduces restriction of daily activities in women with severe diarrhoea-predominant IBS. Aliment Pharmacol Ther 2012;36:437–48 22779693 10.1111/j.1365-2036.2012.05208.x PMC3464357

[39] Spiegel B Strickland A Naliboff BD Predictors of patient-assessed illness severity in irritable bowel syndrome. Am J Gastroenterol 2008;103:2536–43 18637089 10.1111/j.1572-0241.2008.01997.x PMC2949074

[40] Olden K DeGarmo RG Jhingran P Patient satisfaction with alosetron for the treatment of women with diarrhea-predominant irritable bowel syndrome. Am J Gastroenterol 2002;97:3139–46 12492201 10.1111/j.1572-0241.2002.07111.x

