# Supplementary material for: A randomised trial of ondansetron for the treatment of irritable bowel syndrome with diarrhoea
Source: Gut. 2013 Dec 12;63(10):1617–25. doi: 10.1136/gutjnl-2013-305989 (PMC4173656; doi:10.1136/gutjnl-2013-305989)
Supplement: Web appendix [file gutjnl-2013-305989-s1.pdf]

Appendix 1

Association between Serotonin transporter promoter polymorphisms and response to treatment

Table of contents

	Pages
Methods	2
Results	2
Table S1	3

Methods

DNA was extracted from 200 µl of citrated blood using the QIAamp DNA Blood Mini Kit (Qiagen Cat. No. 51106) and then genotyped. PCR included DMSO and used the primer sequences FORWARD 5'GGCGTTGCCGCTCTGAATGC3' and REVERSE 5'GAGGGACTGAGCTGGACAACCAC3'. We used biotaq (bioline) 54°C and 35 cycles. PCR products were run on a 2% agarose gel to distinguish the difference in band sizes of 484/528bp. A band of 528bp indicated *l/l*, 484 and 528bp *l/s* and 484bp only indicated *ss* genotype. The alleles were in Hardy Weinberg equilibrium.

The differences across the groups were tested with ANOVA.

Results

Genotype data are available for 96 (97%) of ITT patients. Thirty (30.3%) patients had *ll* genotype, 25 (25.3%) had *sl* genotype and 41 (41.4%) had *ss* genotype. No statistically significant differences across the groups were found for primary or most secondary outcomes (Table S1). However our data indicate that *sl* genotype patients may react more favorably to Ondansetron (bigger improvement in stool form and bigger increase in transit time) compared to ll genotype patients though the differences never reached 5% statistical significance (data on request).

Table S1. Mean differences (95% confidence intervals) between Ondansetron and placebo periods for various genotypes[1,2]

	Allele types		
	ll	*sl*	*ss*
Primary and secondary efficacy parameters (differences between Ondansetron placebo periods)			
Stool form	-0.7 (-1.2, -0.3)**	-1.2 (-1.6, -1.8)***	-0.5 (-1.0, 0.0)
p-value		*0.069*	
Days with pain	-0.1 (-0.8, 0.7)	-0.4 (-1.1, 0.2)	-0.3 (-1.2, 0.5)
p-value		*0.741*	
Days with urgency	-0.7 (-1.4, 0.1)	-1.2 (-1.9, -0.5)**	-1.3 (-2.3, -0.5)**
p-value		*0.467*	
Days with bloating	-0.6 (-1.3, 0.2)	-0.5 (-1.1, 0.2)	-1.1 (-2.0, -0.3)**
p-value		*0.421*	
Pain score	-0.07 (0.29, 0.15)	-0.12 (-0.31, 0.07)	-0.09 (-0.34, 0.15)
p-value		*0.943*	
Urgency score	-0.20 (-0.44, 0.04)	-0.39 (-0.59, -0.19)***	-0.35 (-0.61, -0.09)**
p-value		*0.472*	
Bloating score	-0.06 (-0.31, 0.19)	-0.07 (-0.28, 0.15)	-0.32 (-0.60, -0.05)*
p-value		*0.280*	
Stool frequency, reduction %	4 (16, -8)	18 (28, 8)**	7 (20, -6)
p-value		*0.177*	
Whole gut transit time increase[3], hours	11.8 (4.5, 19.1)**	17.1 (10.6, 23.7)***	4.9(-3.0, 12.8)
p-value		*0.066*	

[1] exact p-values shown for the overall difference across the genotypes

[2]Statistical significance indicated for tests of the mean differences against 0.

[3]The data are available for 87 patients

*p<0.05, **p<0.01, ***p<0.001
